# High-flow nasal cannula oxygen therapy versus noninvasive ventilation in immunocompromised patients with acute respiratory failure: an observational cohort study

**DOI:** 10.1186/s13613-016-0151-7

**Published:** 2016-05-20

**Authors:** Rémi Coudroy, Angéline Jamet, Philippe Petua, René Robert, Jean-Pierre Frat, Arnaud W. Thille

**Affiliations:** Service de Réanimation Médicale, CHU de Poitiers, 2, rue de la Milétrie, 86021 Poitiers, France; INSERM CIC 1402 (ALIVE Group), Université de Poitiers, Poitiers, France

**Keywords:** Acute respiratory failure, Immunosuppression, Noninvasive positive pressure ventilation, Acute lung injury, Mechanical ventilation, High-flow oxygen therapy

## Abstract

**Background:**

Acute respiratory failure is the main cause of admission to intensive care unit in immunocompromised patients. In this subset of patients, the beneficial effects of noninvasive ventilation (NIV) as compared to standard oxygen remain debated. High-flow nasal cannula oxygen therapy (HFNC) is an alternative to standard oxygen or NIV, and its use in hypoxemic patients has been growing. Therefore, we aimed to compare outcomes of immunocompromised patients treated using HFNC alone or NIV as a first-line therapy for acute respiratory failure in an observational cohort study over an 8-year period. Patients with acute-on-chronic respiratory failure, those treated with standard oxygen alone or needing immediate intubation, and those with a do-not-intubate order were excluded.

**Results:**

Among the 115 patients analyzed, 60 (52 %) were treated with HFNC alone and 55 (48 %) with NIV as first-line therapy with 30 patients (55 %) receiving HFNC and 25 patients (45 %) standard oxygen between NIV sessions. The rates of intubation and 28-day mortality were higher in patients treated with NIV than with HFNC (55 vs. 35 %, p = 0.04, and 40 vs. 20 %, p = 0.02 log-rank test, respectively). Using propensity score-matched analysis, NIV was associated with mortality. Using multivariate analysis, NIV was independently associated with intubation and mortality.

**Conclusions:**

Based on this observational cohort study including immunocompromised patients admitted to intensive care unit for acute respiratory failure, intubation and mortality rates could be lower in patients treated with HFNC alone than with NIV. The use of NIV remained independently associated with poor outcomes.

**Electronic supplementary material:**

The online version of this article (doi:10.1186/s13613-016-0151-7) contains supplementary material, which is available to authorized users.

## Background

Acute respiratory failure is the main cause of admission to intensive care unit (ICU) in immunocompromised patients [1]. In this subset of patients, the need for intubation and invasive mechanical ventilation is associated with particularly high mortality rates, reaching 70 % of cases [2–4]. In the early 2000s, two randomized controlled trials reported lower rates of intubation and mortality with the use of noninvasive ventilation (NIV) as compared to standard oxygen [5, 6]. However, given the small samples of patients included in these studies, experts suggested that NIV could be used in immunocompromised patients with acute respiratory failure, but the strength of recommendation was assessed as weak [7]. As a consequence, so far NIV has been used as a first-line therapy in only 25–40 % of immunocompromised patients admitted to ICU for acute respiratory failure [1, 8–10]. Recently, a large randomized controlled trial did not confirm the potential benefits of NIV and in fact found similar outcomes in immunocompromised patients with acute respiratory failure treated with NIV or oxygen alone [11]. It is important to note that, in this study, oxygen therapy could be delivered using either standard oxygen or high-flow oxygen through nasal cannula (HFNC).

HFNC is a recent technique that delivers heated and humidified oxygen at high-flow rates [12]. Several physiological studies have shown HFNC to be better tolerated than standard oxygen delivered through a mask [13–15]. High-flow rates of fresh gas help to increase the fraction of inspired oxygen (FiO_2_) [16], to generate low levels of positive end-expiratory pressure [17], and to decrease physiological dead space by flushing expired carbon dioxide in the upper airways [18]. The result is a decrease in work of breathing [19] and dyspnea [14] while the heating and humidification of inspired gases may prevent thick secretions and atelectasis. HFNC could not only offer an alternative to standard oxygen in hypoxemic patients, but also avoid the need for NIV. In a recent multicenter randomized controlled trial, the mortality rate in patients with acute respiratory failure treated with HFNC alone was significantly lower in both those treated with standard oxygen and in those treated with NIV [20]. In this study, patients treated with NIV also received HFNC between NIV sessions, thereby suggesting a direct deleterious effect of NIV compared to the group receiving HFNC alone. That said, as patients with neutropenia were excluded from the trial, these results could not be extrapolated to all immunocompromised patients.

Given the fact that use of HFNC in patients with acute respiratory failure has been increasing in our unit over recent years, we aimed to compare the outcomes of immunocompromised patients treated with HFNC alone or with NIV as first-line therapy.

Some of the results of this study were reported in the form of an abstract at the 2016 meeting of the French Intensive Care Society in Paris, France.

## Methods

### Study design

Between 1 January 2007 and 31 December 2014, discharge reports from all patients admitted to our 15-bed medical ICU in a tertiary hospital were retrospectively reviewed. This study was approved by the Ethics Committee of the French Intensive Care Society (*Société de Réanimation de Langue Française*, SRLF, CE no. 14-27), and given its observational nature, informed consent was waived.

### Screening of patients

We screened all patients admitted for acute respiratory failure defined by the following criteria: a respiratory rate ≥25 breaths/min or clinical signs of respiratory distress, and a calculated PaO_2_-to-FiO_2_ ratio ≤300 mmHg, FiO_2_ being estimated as follows: (oxygen flow in liters per minute × 0.03) + 0.21 [20]. Among them, we included those who had immunosuppression caused by hematologic or solid cancer, stem cell or solid organ transplantation, a steroid dose of more than 0.5 mg/kg for at least 1 month, or cytotoxic drugs for non-malignant disease or acquired immune deficiency syndrome. Patients with acute-on-chronic respiratory failure, those treated with standard oxygen alone or needing immediate intubation, and those with a do-not-intubate order were excluded from the analysis.

### Classification of patients

Patients were classified according to the time from the onset of acute respiratory failure and the start of the first-line strategy of ventilatory support including NIV or HFNC. All patients in whom NIV was started within the first 6 h after the onset of acute respiratory failure were included in the NIV group if they received at least 2 h of NIV within the first 24 h. Those who were treated with HFNC within the first 6 h after the onset of acute respiratory failure were included in the HFNC group, even if they received late NIV as a rescue therapy beyond the first 6 h. Therefore, patients initially treated with HFNC and who received late NIV as rescue therapy, i.e., the most severe patients, remained classified in the HFNC group. We excluded patients treated with standard oxygen during the first 6 h and who received short NIV (<2 h) considered as preoxygenation in case of frank respiratory worsening leading to intubation, and those treated with standard oxygen during the first 6 h and who received late NIV as rescue therapy. Each patient was classified by consensus of three senior intensivists (RC, JPF, and AWT) blinded to outcomes up to full agreement.

In our unit, the criteria to decide intubation were the same as those used in our previous studies [15, 20]: uncontrolled shock defined by mean arterial pressure ≤65 mm Hg despite a 30 ml/kg crystalloid fluid challenge and increasing doses of vasopressors, neurological impairment defined by a Glasgow score ≤12, or signs of persisting or worsening respiratory failure as defined by at least two of the following criteria: respiratory rate >40 breaths per minute, lack of improvement in signs of high respiratory muscle workload, development of copious tracheal secretions, acidosis with pH <7.35, an SpO_2_ <90 % for more than 5 min without technical dysfunction, or a poor response to oxygenation techniques.

### Data collection

 For all included patients, we collected age, gender, functional status before ICU admission using the Knaus chronic health status score [21], Mac Cabe score reflecting the severity of underlying disease [22], severity scores including the Simplified Acute Physiology Score II [23], and the modified Sequential Organ Failure Assessment (excluding respiratory item) [24], type of immunosuppression, and year of ICU admission. Clinical, radiological, and biological parameters at inclusion such as heart rate, systolic blood pressure, respiratory rate, SpO_2_, body temperature, bilateral lung infiltrates on chest X-ray, arterial pH, sodium bicarbonate, and PaO_2_-to-FiO_2_ ratio were recorded. Two senior physicians reviewed all charts to assess the reason for acute respiratory failure (AJ and PP). Initial settings during NIV or HFNC and ventilation characteristics during the ICU stay were collected.

### Outcomes

Primary end-point was the mortality rate at day 28. Secondary outcomes included intubation rate, length of mechanical ventilation and ICU stay, in-ICU mortality, and variables associated with intubation and mortality at day 28.

### Statistical analysis

Continuous variables were expressed as mean ± standard deviation (SD) or as median [interquartile range, from 25th to 75th percentiles] according to their distribution using the Kolmogorov–Smirnov test and compared using the Mann–Whitney or the Student’s *t* test as appropriate. Dichotomous variables were expressed in percentage and compared using the Fischer’s exact test or the Chi-square test as appropriate. We performed two multivariate analyses using a backward step-down logistic regression model including early clinical and biological variables associated first with mortality at day 28 and second with intubation, with a p value <0.15 using univariate analysis. As the year of ICU admission was different between the 2 groups, this variable was forced in the logistical regression model. Kaplan–Meier curves were plotted to assess time from the onset of acute respiratory failure to mortality within the first 28 days in the 2 groups and compared by the log-rank test. Given the baseline differences between groups, a propensity score was computed by using logistic regression with the dependent variables associated with mortality at day 28 (age and use of vasopressors within 24 h after ICU admission) to estimate the effect of NIV on mortality at day 28 [25]. A matching algorithm was performed according to the propensity score. Adjusted outcomes between patients who were or were not treated with NIV were compared using the paired t test or the Wilcoxon matched paired test as appropriate to compare adjusted outcomes. We considered two-tailed p values <0.05 as significant. Statistical analyses were performed using the statistical software package XLstat^®^ (Addinsoft, Paris, France), GraphPadPrism 5^®^ (La Jolla, CA, USA) and R statistical package (online at http://www.R-project.org).

## Results

**Fig. 1:**
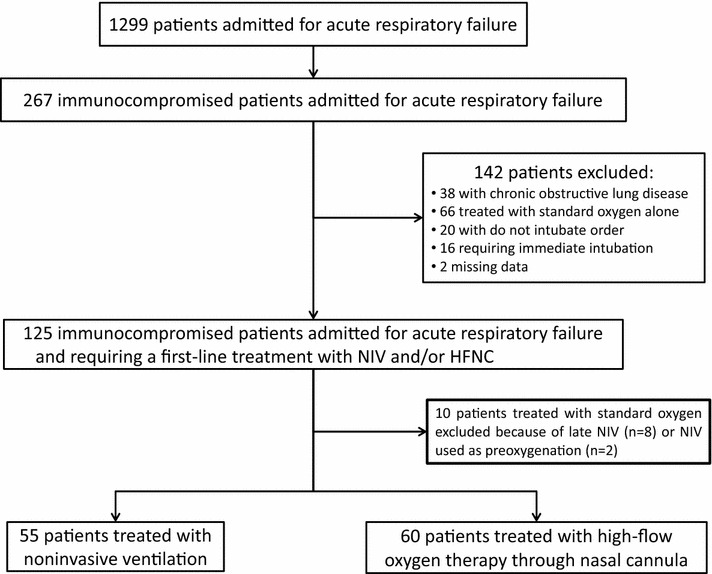
Flow chart of included patients over an 8-year period

Of the 5244 patients admitted to our unit over an 8-year period, 1299 (25 %) were admitted for acute respiratory failure. Among them, 267 (21 %) were immunocompromised (Fig. 1). Baseline characteristics of the 115 patients (43 %) included in the analysis are given in Table 1. In the NIV group, patients were more likely to be male, to have hypercapnia and alkalemia at admission, whereas in the HFNC group they tended to be older. In the first half of the study period, patients were more likely to be treated with NIV as first-line therapy than in the second half: 68 % (26 of 38 patients) received NIV from 2007 versus 2010 versus 38 % (22 of the 77 patients) from 2011 to 2014, p = 0.003. Intubation rates in the NIV group did not differ between the 2 periods: 57 % (15/26 patients) in the first period versus 52 % (15/29) in the second one (p = 0.66).

**Table 1:** Comparison of baseline characteristics and outcomes between patients treated by noninvasive positive pressure ventilation (NIV) or high-flow nasal cannula (HFNC) oxygen therapy alone

	NIV (*n* = 55)	HFNC (*n* = 60)	*p* value
Age (years)	58 (44–66)	62 (50–70)	0.06
Gender (male)	42 (76 %)	35 (58 %)	0.048
Knaus chronic health status score			0.46
A	15 (27 %)	19 (32 %)	
B	17 (31 %)	22 (37 %)	
C	21 (38 %)	15 (25 %)	
D	2 (3.6 %)	4 (6.7 %)	
Mac Cabe classification			0.20
1	20 (36 %)	19 (32 %)	
2	19 (35 %)	30 (50 %)	
3	16 (29 %)	11 (18 %)	
SAPS II at ICU admission (points)	42 ± 11	46 ± 13	0.10
Modified SOFA score at inclusion (points)	3 (1–6)	4 (1–6)	0.28
Type of immunosuppression			0.30
Hematologic cancer or neutropenia	33 (60 %)	31 (52 %)	
Solid cancer	11 (20 %)	8 (13 %)	
Drug-induced immunosuppression	10 (18 %)	20 (33 %)	
Acquired immune deficiency syndrome	1 (2 %)	1 (2 %)	
Cause of respiratory failure			0.38
Documented infection	24 (44 %)	31 (52 %)	
Cardiogenic pulmonary edema	5 (9 %)	5 (8 %)	
Specific	13 (24 %)	6 (10 %)	
Other identified causes	7 (13 %)	11 (18 %)	
Not identified cause	6 (11 %)	7 (12 %)	
Clinical and biological parameters at inclusion			
Heart rate (beats/min)	111 ± 22	113 ± 23	0.71
Systolic blood pressure (mmHg)	130 (113–150)	119 (110–147)	0.28
Respiratory rate (breaths/min)	30 (26–33)	29 (26–32)	0.75
SpO_2_ (%)	94 (91–98)	96 (94–99)	0.02
Body temperature (°C)	37.8 ± 1.1	38.0 ± 1.1	0.47
Bilateral lung infiltrates on chest X-ray	46 (84 %)	50 (83 %)	>0.99
Arterial pH	7.44 (7.40–7.47)	7.46 (7.43–7.50)	0.02
PaO_2_-to-FiO_2_ ratio (mmHg)	141 (111–177)	149 (107–204)	0.19
PaO_2_-to-FiO_2_ ratio ≤ 200 mmHg	47 (85 %)	44 (73 %)	0.17
PaCO_2_ (mmHg)	37 (32–45)	32 (29–38)	<0.0001
PaCO_2_ > 45 mmHg	12 (22 %)	2 (3 %)	0.003
Sodium bicarbonate (mmol/l)	25 (22–28)	21 (24–26)	0.04
Vasopressors within 24 h after ICU admission	9 (16 %)	14 (23 %)	0.35
Time from admission to ventilatory support initiation (h)	1 (0–1)	1 (0–1)	0.62
Need for immunosuppressive drug during ICU stay	13 (24 %)	15 (25 %)	>0.99
Admission before 2011	26 (47 %)	12 (20 %)	<0.0001
Primary outcome			
28-day mortality	22 (40 %)	12 (20 %)	0.02
Secondary outcomes			
Intubation	30 (55 %)	21 (35 %)	0.04
Time from admission to intubation (h)	28 (18–49)	35 (9–49)	0.99
Length of invasive mechanical ventilation (days)	8 (4–11)	7 (4–12)	0.63
Length of ICU stay (days)	8 (5–13)	7 (4–9)	0.08
In-ICU mortality	20 (36 %)	9 (15 %)	0.01

Nominal variables are given as number (%), and continuous data are given as median (25th–75th percentile) or mean ± standard deviation (SD) according to their distribution using the Kolmogorov–Smirnov test

*SAPS* Simplified Acute Physiology Score, *SOFA* Sequential Organ Failure Assessment

In the NIV group, initial FiO_2_ was 0.6 [0.5–0.9], whereas levels of pressure support and positive end-expiratory pressure were 10 cm H_2_O [8–12] and 4 cm H_2_O [4–5], respectively. Mean expiratory tidal volume delivered during the first 24 h after NIV initiation was 9.0 ± 2.4 ml/kg of predicted body weight. NIV was applied during 2.0 days [1.0–4.0] in median for a duration of 8 h [4–11] during the first 24 h. Among the 55 patients treated with NIV, 25 patients (45 %) received standard oxygen between NIV sessions, whereas the 30 other patients (55 %) received HFNC.

In the HFNC group, FiO_2_ was 0.6 [0.5–1], whereas gas flow was 50 l/min [40–50]. HFNC was applied continuously for a total duration of 2.0 days [1.0–4.0] in median. Eight patients in the HFNC group (13 %) received NIV as rescue therapy during their ICU stay.

**Fig. 2:**
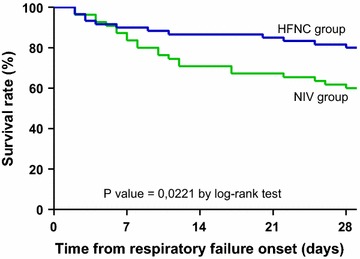
Figure showing the Kaplan–Meier plots of the cumulative survival rates within the 28 days following the onset of acute respiratory failure in ICU in the overall population. The rate of mortality was significantly lower in patients treated with high-flow nasal cannula (HFNC) oxygen therapy alone (*blue line*) than in patients treated with noninvasive ventilation (NIV) as first-line therapy (*green line*), decreasing from 40 % (22/55) to 20 % (12/60) *p* = 0.0221 by log-rank test

Overall intubation rate was 44 % (51 of 115 patients), and overall mortality at day 28 was 30 % (34 of 115 patients). The rates of intubation and of mortality in ICU and at day 28 were significantly lower in the HFNC group than in the NIV group (Table 1 and Fig. 2). Mortality of patients who needed intubation tended to be significantly lower in the HFNC group (9/21 patients, 43 %) than in the NIV group (21/30 patients, 70 %, p = 0.05).

In the NIV group, outcomes did not significantly differ between the patients who received HFNC between NIV sessions and those who received standard oxygen: the rates of intubation were 47 % (14/30) versus 64 % (16/25), respectively, p = 0.28; the rates of mortality at day 28 were 36 % (11/30) versus 44 % (11/25), respectively, p = 0.59.

Variables associated with intubation and mortality at day 28 in the overall population are given in Additional file 1 and Table 2, respectively. Using multivariate analysis, the 3 variables independently associated with intubation were severity at admission in the ICU as indicated by a high SAPS II, need for vasopressor within the 24 h after ICU admission, and use of NIV (Table 3). Use of NIV remained associated with mortality at day 28 independently from age and the need for vasopressor within 24 h after ICU admission (Table 3), even after forcing the year of admission in the model.

**Table 2:** Univariate analysis of variables associated with mortality at day 28 in the overall population

	Survivors (*n* = 81)	Non-survivors (*n* = 34)	Odds ratio (95 % CI)	*p* value
Demographic variables				
Age (years)	56 ± 15	60 ± 15	1.02 (0.99–1.05)	0.13
Gender (male)	54 (67 %)	23 (68 %)	0.96 (0.41–2.25)	0.92
ICU admission before 2011	24 (30 %)	14 (41 %)	1.66 (0.72–3.82)	0.23
Noninvasive ventilation as a first-line therapy	34 (42 %)	22 (65 %)	2.67 (1.16–6.12)	0.03
SAPS II score	42 ± 12	47 ± 13	1.036 (1.00–1.07)	0.04
Modified SOFA score excluding respiratory item	3 (1–6)	4 (1–7)	1.06 (0.93–1.21)	0.44
Knaus chronic health status score				0.32
A	24 (30 %)	10 (29 %)	1	
B	31 (38 %)	8 (24 %)	0.62 (0.21–1.81)	
C	23 (28 %)	13 (38 %)	1.36 (0.50–3.70)	
D	3 (3.7 %)	3 (8.8 %)	2.40 (0.41–13.98)	
Mac Cabe classification				0.32
1	28 (35 %)	11 (32 %)	1	
2	37 (46 %)	12 (35 %)	0.83 (0.32–2.14)	
3	16 (20 %)	11 (32 %)	1.75 (0.62–4.94)	
Type of immunosuppression				0.08
Hematologic cancer or neutropenia	49 (60 %)	15 (44 %)	1	
Solid cancer	9 (11 %)	10 (29 %)	3.53 (1.21–10.27)	
Drug-induced immunosuppression	21 (26 %)	9 (26 %)	1.41 (0.54–3.70)	
Acquired immune deficiency syndrome	2 (2 %)	0 (0 %)	0.64 (0.02–27.52)	
Variables at inclusion				
Heart rate (bpm)	111 ± 23	113 ± 21	1.00 (0.99–1.02)	0.71
Systolic arterial blood pressure (mmHg)	126 ± 24	126 ± 29	1.00 (0.98–1.02)	0.96
Diastolic arterial blood pressure (mmHg)	67 ± 18	67 ± 13	1.00 (0.98–1.02)	0.99
Respiratory rate (breaths/min)	28 (25–32)	30 (27–34)	1.04 (0.98–1.10)	0.19
SpO_2_ (%)	96 (94–99)	94 (90–97)	0.90 (0.83–0.97)	0.01
Body temperature (°C)	38.0 ± 1.2	37.7 ± 0.9	0.82 (0.58–1.18)	0.29
pH	7.46 (7.42–7.49)	7.44 (7.40–7.49)	0.59 (0.00–21.07)	0.30
Sodium bicarbonate (mmol/l)	24 (22–27)	24 (22–27)	1.03 (0.94–1.12)	0.83
PaO_2_-to-FiO_2_ ratio (mmHg)	158 ± 59	141 ± 48	0.99 (0.99–1.00)	0.15
PaO_2_-to-FiO_2_ ratio ≤ 200 mmHg	62 (77 %)	29 (85 %)	1.78 (0.60–5.23)	0.29
PaO_2_ (mmHg)	74 (61–88)	70 (57–93)	1.00 (0.99–1.01)	0.38
PaCO_2_ (mmHg)	34 (31–40)	34 (31–45)	1.01 (0.97–1.06)	0.56
PaCO_2_ > 45 mmHg	8 (10 %)	6 (18 %)	1.96 (0.62–6.14)	0.24
Bilateral lung infiltrate	69 (85 %)	27 (79 %)	0.67 (0.24–1.89)	0.45
Vasopressors within 24 h after ICU admission	10 (12 %)	13 (38 %)	2.18 (0.85–5.62)	0.11
Immunosuppressive drug during ICU stay	22 (27 %)	6 (17 %)	0.58 (0.21–1.58)	0.35
Cause of respiratory failure				0.13
Documented infection	38 (47 %)	17 (50 %)	1	
Cardiogenic pulmonary edema	10 (12 %)	0 (0 %)	0.11 (0.01–2.17)	
Specific	11 (14 %)	8 (24 %)	1.63 (0.56–4.76)	
Other identified causes	12 (15 %)	6 (18 %)	1.14 (0.37–3.54)	
Not identified cause	10 (12 %)	3 (9 %)	0.73 (0.19–2.91)	

Nominal variables are given as number (%), and continuous data are given as median (25th–75th percentile) or mean ± standard deviation (SD) according to their distribution using the Kolmogorov–Smirnov test

*SAPS* Simplified Acute Physiology Score, *SOFA* Sequential Organ Failure Assessment

**Table 3:** Multivariate analysis of variables associated with outcomes in the overall population

	Adjusted odds ratio (95 % CI)	*p* value
*Variables independently associated with intubation* ^a^		
Simplified Acute Physiology Score II, per point	1.04 (1.00–1.08)	0.04
Noninvasive ventilation as a first-line therapy	3.25 (1.39–7.60)	0.007
Use of vasopressors within 24 h after ICU admission	4.12 (1.32–12.84)	0.02
*Variables independently associated with mortality at day 28* ^b^		
Age (per year)	1.03 (1.00–1.07)	0.04
Use of vasopressors within 24 h after ICU admission	2.83 (1.02–7.91)	0.047
Noninvasive ventilation as a first-line therapy	3.70 (1.49–9.19)	0.005

^a^Non-collinear variables included in the logistical regression model were Simplified Acute Physiology Score II, Noninvasive ventilation as a first-line therapy, use of vasopressors within 24 h after ICU admission, SpO_2_ at ICU admission, cause of respiratory failure and PaCO_2_ as a continuous variable. The year of ICU admission was forced in the model

^b^Non-collinear variables included in the logistical regression model were age, PaO_2_-to-FiO_2_ ratio at ICU admission, use of noninvasive ventilation as a first-line therapy, type of immunosuppression, use of vasopressors in the 24 h after ICU admission, cause of respiratory failure and PaCO_2_ > 45 mmHg. The year of ICU admission was forced in the model

Baseline characteristics and outcomes of the 57 patients included in the propensity score-matched cohort are displayed in Table 4. In-ICU mortality at day 28 remained significantly lower in the HFNC than in the NIV group after matching on age and need for vasopressors within 24 h after ICU admission (Table 4). Using multivariate analysis in the matched cohort, NIV as a first-line therapy was the only factor independently associated with mortality at day 28 with and adjusted odds ratio of 4.03 and a 95 % confidence interval of [1.09–14.93], even after forcing the year of ICU admission.

**Table 4:** Comparison of baseline characteristics and outcomes between propensity score-matched patients treated by noninvasive positive pressure ventilation (NIV) or high-flow nasal cannula (HFNC) oxygen therapy alone

	NIV (*n* = 24)	HFNC (*n* = 33)	*p* value
Age (years)	62 ± 11	62 ± 11	0.72
Gender (male)	18 (75 %)	17 (52 %)	0.13
Knaus chronic health status score			0.53
A	8 (33 %)	9 (27 %)	
B	6 (25 %)	11 (33 %)	
C	10 (42 %)	11 (33 %)	
D	0 (0.0 %)	2 (6.1 %)	
Mac Cabe classification			0.27
1	11 (46 %)	12 (36 %)	
2	6 (25 %)	15 (45 %)	
3	7 (29 %)	6 (18 %)	
SAPS II at ICU admission (points)	40 ± 11	44 ± 12	0.52
Modified SOFA score at inclusion (points)	1.5 (0.0–4.0)	3.0 (1.0–6.0)	0.44
Type of immunosuppression			0.19
Hematologic cancer or neutropenia	12 (50 %)	18 (55 %)	
Solid cancer	7 (29 %)	3 (9.1 %)	
Drug-induced immunosuppression	5 (21 %)	11 (33 %)	
Acquired immune deficiency syndrome	0 (0.0 %)	1 (3.0 %)	
Cause of respiratory failure			0.08
Documented infection	9 (38 %)	19 (58 %)	
Cardiogenic pulmonary edema	4 (27 %)	3 (9.1 %)	
Specific	6 (25 %)	1 (3.0 %)	
Other identified causes	2 (8.3 %)	6 (18 %)	
Not identified cause	3 (13 %)	4 (12 %)	
Clinical and biological parameters at inclusion			
Heart rate (beats/min)	107 ± 21	112 ± 21	0.55
Systolic blood pressure (mmHg)	140 ± 22	127 ± 23	0.17
Respiratory rate (breaths/min)	30 ± 6	29 ± 6	0.76
SpO_2_ (%)	94 ± 5	96 ± 4	0.10
Body temperature (°C)	37.9 ± 1.1	37.9 ± 1.1	0.66
Bilateral lung infiltrates on chest X-ray	19 (79 %)	31 (93 %)	0.12
Arterial pH	7.45 ± 0.07	7.46 ± 0.06	0.43
PaO_2_-to-FiO_2_ ratio (mmHg)	154 ± 57	156 ± 57	0.98
PaO_2_-to-FiO_2_ ratio ≤ 200 mmHg	18 (75 %)	24 (73 %)	0.85
PaCO_2_ (mmHg)	39 ± 8	33 ± 5	0.03
PaCO_2_ > 45 mmHg	4 (17 %)	1 (3.0 %)	0.15
Sodium bicarbonate (mmol/l)	26 ± 4	24 ± 4	0.10
Vasopressors within 24 h after ICU admission	1 (4.2 %)	4 (12 %)	0.39
Time from admission to ventilatory support initiation (h)	1 (0–1)	1 (0–1)	0.98
Need for immunosuppressive drug during ICU stay	5 (21 %)	4 (12 %)	0.47
Admission before 2011	12 (50 %)	7 (21 %)	0.04
Primary outcome			
28-day mortality	10 (42 %)	5 (15 %)	0.03
Secondary outcomes			
Intubation	13 (54 %)	10 (30 %)	0.07
Mortality of intubated	10/13 (77 %)	4/10 (40 %)	0.07
Time from admission to intubation (h)	48 (20–78)	35 (22–59)	>0.99
Length of invasive mechanical ventilation (days)	8 (5–18)	5 (3–10)	>0.99
Length of ICU stay (days)	7 (5–16)	6 (4–9)	0.13
In-ICU mortality	10 (42 %)	4 (12 %)	0.01

Nominal variables are given as number (%), and continuous data are given as median (25th–75th percentile) or mean ± standard deviation (SD) according to their distribution using the Kolmogorov–Smirnov test

*SAPS* Simplified Acute Physiology Score, *SOFA* Sequential Organ Failure Assessment

## Discussion

Our main finding is that immunocompromised patients admitted to ICU for acute respiratory failure had higher mortality when treated with NIV than those treated with HFNC alone. Moreover, they were more likely to be intubated and to have prolonged ICU length of stay. After adjustment, NIV remained independently associated with intubation and mortality at day 28.

In our study, intubation and mortality rates in the NIV group of the overall cohort were 55 and 40 %, respectively. These results are in keeping with the intubation and mortality rates reported in recent cohort studies [2, 26–28], reinforcing the external validation of our results. Conversely, the rates of intubation and mortality in our patients treated with HFNC alone were 35 and 20 %, respectively, which are markedly lower than the rates reported in the above-mentioned studies [2, 26–28]. Therefore, these differences seem more likely due to a decrease in intubation or mortality rates observed in the HFNC group rather than an excess of intubation or mortality in the NIV group.

In contrast to patients with chronic obstructive pulmonary disease [29, 30] or cardiogenic pulmonary edema [31], the benefits of NIV remain unclear in immunocompromised patients with acute respiratory failure. To date, three randomized controlled trials have compared the use of NIV versus standard oxygen in immunocompromised patients with acute respiratory failure [5, 6, 11]. In a first trial including 40 patients with solid organ transplantation, the rate of intubation was significantly reduced in patients treated with NIV [5]. However, nearly one quarter of the patients had cardiogenic pulmonary edema [5], a condition for which the benefits of NIV are supported by a strong level of evidence [31]. In a second trial including 52 patients, the rates of intubation and mortality were significantly lower in patients treated with NIV [6]. However, these beneficial effects were observed only in patients with hematologic cancer or neutropenia, which accounted only for 15 patients per group [6]. In the most recent trial including 374 patients, intubation and mortality rates did not differ between the two groups [11]. However, respiratory rate and oxygen requirement at inclusion were lower than in our study and in the two previous trials [5, 6], perhaps illustrating a lower severity of respiratory failure, which may have attenuated the impact of NIV on outcomes.

The case volume of patients treated with first-line NIV may also have influence on outcomes, with lower expected intubation rates in highly skilled centers. These findings have already been suggested in patients treated with NIV for cardiogenic pulmonary edema or acute-on-chronic respiratory failure [9, 32–35]. In our high case-volume center, this would have favoured the NIV group and attenuated the outcome difference between the 2 groups, which was not the case.

A recent retrospective study including 178 immunocompromised patients with acute respiratory failure suggested that the best strategy consisted in use of NIV associated with HFNC between NIV sessions [28]. The 37 % mortality rate recorded in the group treated by NIV and HFNC was almost the same as that of our patients treated by NIV (40 %). Once again, this mortality rate remained markedly higher than the 20 % rate we report herein in our patients treated with HFNC alone. Therefore, use of HFNC alone without NIV could be the treatment of choice in immunocompromised patients admitted to ICU for acute respiratory failure.

Although use of HFNC alone has been poorly evaluated in immunocompromised patients with acute respiratory failure, our results are in line with those found recently in a large multicenter randomized controlled trial [20]. Indeed, this study showed a significantly reduced mortality rate in patients treated with HFNC alone as compared to those treated by NIV with HFNC between NIV sessions [20]. In this trial, about one-third of included patients were immunocompromised, and the rates of intubation and in-ICU mortality in the HFNC group were 38 and 11 %, respectively, which are in line with those we report.

The beneficial effects of HFNC could be largely due to tolerance. HFNC seems better tolerated than NIV in patients with acute respiratory failure with a higher degree of comfort, a reduction in the severity of dyspnea and a decreased respiratory rate [15, 20]. Although these criteria were not assessed in our study, we believe that our findings may be extrapolated to immunocompromised patients. By contrast, NIV could be harmful due to potential ventilator-induced lung injury generated by pressure support that increases tidal volumes [36] and leads to high transpulmonary pressure [37]. Indeed, it is well established that mortality of patients with acute respiratory distress syndrome (ARDS) is lower using low tidal volumes approximating 6 ml/kg of predicted body weight [38]. Even in patients without criteria for ARDS, the use of low tidal volumes may reduce the risk of developing ARDS [39]. In our study, the majority of patients treated with NIV had clinical criteria for ARDS according to the recent definition [40], and the expiratory tidal volumes delivered to these patients under NIV were around 9.0 ml/kg of predicted body weight. Although such high volumes are similar to those reported in recent studies focusing on NIV in acute respiratory failure [20, 34], they could be particularly deleterious by worsening lung injury. Indeed, in the study by Carteaux and colleagues, an expired tidal volume above 9.5 ml/kg of predicted body weight was a strong predictor of NIV failure in hypoxemic patients [36]. Despite the absence of expired tidal volume assessment in the HFNC group, the higher intubation rate observed in the NIV group may be explained by the high proportion of patients with an expired tidal volume above 9.5 ml/kg of predicted body weight (46 % of the patients treated with NIV). In addition, any potential deleterious effect of delayed intubation in patients treated with NIV [41, 42] can be ruled out as time from ICU admission to intubation was not longer than in patients treated with HFNC alone.

### Limitations

Our study has several limitations. First, the study was monocentric and performed in a unit with experience in noninvasive management of immunocompromised patients with acute respiratory failure. Indeed, each year about 15 immunocompromised patients are treated with first-line noninvasive ventilatory support, which is close to the number of patients admitted in other highly skilled centers [2, 26]. Therefore, these results could not be extrapolated to centers with less experience. Second, the retrospective nature of the study might have induced selection bias despite the careful classification of patients included in the analysis. Indeed, the baseline characteristics of patients were not similar as patients in the HFNC group were older and as there was a higher proportion of patients with respiratory acidosis in the NIV group. NIV could have been preferentially used in hypercapnic patients due to its efficacy in correction of alveolar hypoventilation [43]. Therefore, the most severe patients might have been more frequently treated with NIV than with HFNC alone. Nevertheless, functional status before ICU admission and baseline severity scores were similar between the two groups. Our intubation and mortality rates in the NIV group were similar to those reported in the literature [2, 6, 26, 27], thereby reinforcing the external validity of our results. Third, it is possible that outcomes of immunocompromised patients admitted to our ICU over this 8-year period had improved in the recent years [44]. However, even after forcing the year of ICU admission in the logistic regression model, NIV remained associated with intubation and mortality. Obviously, these results do not allow for definitive conclusion on the deleterious effects of NIV in this population, and our findings need to be confirmed in a randomized trial.

## Conclusion

Based on this retrospective cohort study, the use of high-flow oxygen therapy through nasal cannula alone may be associated with better outcomes than noninvasive ventilation in immunocompromised patients admitted to intensive care unit for acute respiratory failure.

## Additional file

10.1186/s13613-016-0151-7 Univariate analysis of variables associated with intubation in the overall population.

## Abbreviations

HFNC: high-flow oxygen therapy through nasal cannula
ICU: intensive care unit
NIV: noninvasive ventilation
