# Supplementary material for: High-flow nasal cannula oxygen therapy versus noninvasive ventilation in immunocompromised patients with acute respiratory failure: an observational cohort study
Source: Ann Intensive Care. 2016 May 20;6:45. doi: 10.1186/s13613-016-0151-7 (PMC4875575; doi:10.1186/s13613-016-0151-7)
Supplement: Supplementary file 1 — 10.1186/s13613-016-0151-7 Univariate analysis of variables associated with intubation in the overall population. [file 13613_2016_151_MOESM1_ESM.doc]

**Univariate analysis of variables associated with intubation in the overall population.**

|  | **Not intubated**  **(n=64)** | **Intubated**  **(n=51)** | **Odds Ratio (95% Confidence Interval)** | **P value** |
| --- | --- | --- | --- | --- |
| *Demographic variables* | | | | |
| Age, years | 58 (48-67) | 60 (49-69) | 1.04 (0.99-1.08) | 0.52 |
| Gender, male | 42 (66) | 35 (69) | 0.91 (0.20-4.13) | 0.84 |
| ICU admission before 2011 | 18 (28) | 20 (39) | 1.65 (0.75-3.61) | 0.24 |
| Noninvasive ventilation as a first-line therapy | 25 (39) | 30 (59) | 2.23 (1.05-4.72) | 0.04 |
| SAPS II score | 41 ± 11 | 47 ± 13 | 1.05 (1.01-1.08) | 0.0064 |
| Modified SOFA score excluding respiratory item | 2 (1-6) | 4 (2-7) | 1.16 (1.02-1.31) | 0.02 |
| Knaus chronic health status score |  |  |  | 0.52 |
| A | 19 (30%) | 15 (29%) | 1 |  |
| B | 25 (39%) | 14 (27%) | 0.71 (0.28-1.82) |  |
| C | 17 (27%) | 19 (37%) | 1.42 (0.55-3.63) |  |
| D | 3 (4.7%) | 3 (5.9%) | 1.27 (0.22-7.20) |  |
| Mac Cabe classification |  |  |  | 0.37 |
| 1 | 24 (38%) | 15 (29%) | 1 |  |
| 2 | 28 (44%) | 21 (41%) | 1.20 (0.51-2.83) |  |
| 3 | 12 (34%) | 15 (29%) | 2.00 (0.74-5.42) |  |
| Type of immunosuppression |  |  |  | 0.35 |
| Hematologic cancer or neutropenia | 38 (59) | 26 (51) | 1 |  |
| Solid cancer | 7 (11) | 12 (24) | 2.42 (0.84-6.95) |  |
| Drug induced immunosuppression | 17 (27) | 13 (25) | 1.12 (0.47-2.69) |  |
| Acquired Immune Deficiency Syndrome | 2 (3) | 0 (0) | 0.29 (0.01-12.38) |  |
| *Variables at inclusion* | | | | |
| Heart rate, bpm | 111 ± 24 | 113 ± 20 | 1.01 (0.99-1.02) | 0.59 |
| Systolic arterial blood pressure, mmHg | 128 ± 25 | 125 ± 27 | 1.00 (0.98-1.01) | 0.54 |
| Diastolic arterial blood pressure, mmHg | 67 ± 18 | 66 ± 16 | 0.99 (0.97-1.02) | 0.62 |
| Respiratory rate, breath/min | 28 (26-32) | 30 (26-33) | 1.03 (0.97-1.09) | 0.30 |
| SpO_2_, % | 96 (93-100) | 95 (91-98) | 0.94 (0.88-1.01) | 0.11 |
| Body temperature, °c | 37.9 ± 1.2 | 37.9 ± 1.1 | 0.94 (0.68-1.30) | 0.71 |
| pH | 7.46 (7.42-7.50) | 7.44 (7.40-7.48) | 0.04 (0.00-9.50) | 0.12 |
| Sodium bicarbonate, mmol/l | 25 (22-27) | 24 (20-27) | 0.98 (0.90-1.05) | 0.20 |
| PaO_2_ to FiO_2_ ratio, mmHg | 154 ± 57 | 151 ± 55 | 1.00 (0.99-1.01) | 0.81 |
| PaO_2_ to FiO_2_ ratio ≤ 200 mmHg | 51 (80) | 40 (78) | 0.93 (0.38-2.29) | >0.99 |
| PaO_2_, mmHg | 75 (63-86) | 70 (58-94) | 1.00 (0.99-1.02) | 0.45 |
| PaCO_2_, mmHg | 35 (31-41) | 33 (30-39) | 0.97 (0.93-1.02) | 0.20 |
| PaCO_2_ > 45 mmHg | 7 (11) | 7 (14) | 1.30 (0.42-3.97) | 0.78 |
| Bilateral lung infiltrate | 53 (83) | 43 (84) | 1.12 (0.41-3.02) | >0.99 |
| Vasopressors within 24 hours after ICU admission | 6 (9) | 17 (33) | 4.83 (1.74-13.44) | 0.002 |
| Time from admission to NIV initiation, hours | 1 (1-3) | 1 (1-2) | 1.08 (0.93-1.25) | 0.99 |
| Immunosuppressive drugs during ICU stay | 18 (28%) | 10 (20%) | 0.62 (0.26-1.50) | 0.38 |
| *Cause of respiratory failure* |  |  |  | 0.04 |
| Documented infection | 29 (45) | 26 (51) | 1 | 0.58 |
| Cardiogenic pulmonary oedema | 9 (14) | 1 (2) | 0.12 (0.02-1.05) | 0.06 |
| Specific | 7 (11) | 12 (24) | 1.91 (0.66-5.59) | 0.24 |
| Other identified causes | 13 (20) | 5 (10) | 0.43 (0.14-1.37) | 0.15 |
| Not identified cause | 6 (9) | 7 (14) | 1.30 (0.39-4.37) | 0.67 |

Nominal variables are given as number (%) and continuous data are given as median (25^th^-75^th^ percentile) or mean ± standard deviation (SD) according to their distribution using the Kolmogorov-Smirnov test.

***Abbreviations:*** *SAPS = Simplified Acute Physiology Score; SOFA =* Sequential Organ Failure Assessment
